# Long-Term Ibrutinib Therapy Reverses CD8^+^ T Cell Exhaustion in B Cell Chronic Lymphocytic Leukaemia

**DOI:** 10.3389/fimmu.2019.02832

**Published:** 2019-12-12

**Authors:** Helen M. Parry, Nikhil Mirajkar, Natasha Cutmore, Jianmin Zuo, Heather Long, Marwan Kwok, Ceri Oldrieve, Chris Hudson, Tatjana Stankovic, Shankara Paneesha, Melanie Kelly, Jusnara Begum, Tina McSkeane, Guy Pratt, Paul Moss

**Affiliations:** ^1^Institute of Immunology and Immunotherapy, University of Birmingham, Birmingham, United Kingdom; ^2^Queen Elizabeth Hospital, University Hospitals Birmingham NHS Foundation Trust, Birmingham, United Kingdom; ^3^St James' University Hospital, Leeds Teaching Hospitals Trust, Leeds, United Kingdom; ^4^Institute of Cancer and Genomic Sciences University of Birmingham, Birmingham, United Kingdom; ^5^Faculty of Medicine & Health Sciences, University of Nottingham, Nottingham, United Kingdom; ^6^Heartlands Hospital, University Hospitals Birmingham NHS Foundation Trust, Birmingham, United Kingdom; ^7^Cancer Research UK Clinical Trials Unit, University of Birmingham, Birmingham, United Kingdom

**Keywords:** ibrutinib, chronic lymphocytic leukaemia (CLL), herpes viruses, exhaustion, EBV—epstein-barr virus, CD8 T cells, cytomegalovirus, immunotherapy

## Abstract

Chronic Lymphocytic Leukaemia (CLL) is associated with immune suppression and susceptibility to infection. CD8^+^ T cell numbers are increased and demonstrate elevated expression of PD-1 and impaired function. The mechanisms driving these features of exhaustion are uncertain but are likely to include chronic immune recognition of tumor and/or infectious agents. We investigated the number, phenotype and function of total and virus-specific CD8+ T cells in 65 patients with CLL and 14 patients undergoing long-term ibrutinib therapy (median 21 months). Ibrutinib substantially reduced the number of both CD3+ T cells and CD8+ T cells. Importantly, this was associated with a reduction in PD-1 expression on CD8+ T cells (median 28 vs. 24%; *p* = 0.042) and 3.5 fold increase in cytokine production following mitogen stimulation. The influence of ibrutinib on antigen-specific CD8+ T cell function was assessed by HLA-peptide tetramers and revealed increased IFNγ and TNFα cytokine responses following stimulation with CMV or EBV peptides together with a 55% reduction in the frequency of “inflated” virus-specific CD8+ T cells. These findings reveal that long-term ibrutinib therapy is associated with substantial reversal of T cell exhaustion in B-CLL and is likely to contribute to the reduced infection risk seen in association with this agent.

## Introduction

Chronic lymphocytic leukaemia (CLL) is associated with marked perturbation of the immune system. A range of defects in T cell function are observed including impaired proliferation, cytotoxicity and cytokine production (1). Increased absolute numbers of CD8+ T cells, expanded populations of oligoclonal memory CD8+ T cells (2) and increased expression of immune checkpoint receptors including PD-1 also feature (3). The etiology of these abnormalities is unclear but may include chronic immune stimulation through infection and/or tumour engagement.

Ibrutinib inhibits Bruton Tyrosine Kinase (BTK) activity and has transformed CLL management (4). Ibrutinib has also been confirmed by molecular techniques to irreversibly inhibit interleukin-2 inducible kinase (ITK). ITK plays an important but not indispensable role in the CD4+ Th1 and CD8+ T cell activation signaling cascade, contributing to enhanced proliferation and activation following TCR ligation. In contrast, its role is pivotal and necessary for CD4+ Th2 polarization and function. As such, inhibition of ITK by ibrutinib encourages a skewing towards a Th1 phenotype and has been shown to advantage CD8+ and Th1 T cells, which rely on the redundant resting lymphocyte kinase (RLK) during ITK inhibition. RLK is a signaling kinase which is not inhibited by ibrutinib and provides additional activation of the TCR signaling cascade in the absence of functional ITK. Data on the impact of ibrutinib-induced ITK inhibition within CD8+ T cell populations is currently lacking (5). Understanding the impact of long-term ibrutinib therapy on immune function is an important question and analysis of antigen-specific responses is also currently lacking. Cytomegalovirus (CMV) and Epstein Barr Virus (EBV) are latent herpesvirus infections which infect the majority of the population. CMV and EBV are known to cause “memory inflation,” a term used to describe the expansion of memory CD8+ T cells directed towards the virus and can arise in healthy individuals but also in patients with immune suppression. (2). Expanded populations of CMV-specific CD8+ T cells develop in patients with CLL that are latently infected with the virus.

T cells are known to be dysfunctional in patients with CLL. The term cell exhaustion is used to describe a state of T cell dysfunction that occurs through chronic antigen stimulation and can arise in the context of chronic viral infection or cancer. Exhausted T cells are characterized by the presence of multiple inhibitory receptors, poor proliferation, and cytotoxicity and impaired cytokine secretion (6). Patients with CLL are known to have features of T cell exhaustion with co-expression of CD244, PD-1, and CD160 at high frequencies (1).

Here we examine global and virus-specific T cell phenotype and function in patients with CLL including patients receiving ibrutinib therapy for up to 32 months. Decreased PD-1 expression and increased cytokine responses were observed within the global T cell repertoire following ibrutinib treatment whilst antigen-specific responses also showed increased functional activity and correction of the increased frequency of virus-specific cells.

## Methods

Seventy-nine patients with CLL were recruited [median age 70 (IQR: 63–79)], including 42 patients who had never been treated and 36 who had received chemo-immunotherapy. Of the 36 patients who had previously been treated, 23 were in remission, whilst 13 patients developed relapsed/refractory disease and subsequently were started on ibrutinib therapy, together with 1 patient who was treated with ibrutinib in a front line setting. The 14 patients being treated with ibrutinib, had received up to 32 months of therapy at the time of analysis. Samples were collected immediately prior to starting ibrutinib therapy and then during a subsequent clinic visit which occurred at least 6 months after starting ibrutinib. All patients included in this study were still taking the drug daily at the point the last sample was taken for analysis. Patients characteristics for the total cohort and patient subgroups can be found in (Supplementary Tables 1–4). Nineteen healthy donors were recruited for controls [median age 72 (IQR. 66–80)].

Following ficoll preparation, plasma and PBMCs were extracted, with CMV and EBV serostatus determined by ELISA and immunofluorescence, respectively (7, 8). DNA extraction was then performed on PBMC pellets using GenElute Mammalian Genomic DNA Miniprep kit (Sigma-Aldrich) and HLA typing was assigned using PCR methodology previously described (9).

### Immunophenotypic Analysis of CMV and EBV-Specific CD8+ T Cells

Immunophenotyping was undertaken following APC-conjugated HLA class I tetramer staining of PBMCs at 37°C for 15 min. Details of the tetramers used can be found in Supplementary Table 5. Tetramers were conjugated to APC and a true tetramer response was verified through the lack of background staining by gating all CD3+ T cells, against CD8+ T cells and using a tetramer negative control. Surface staining with the following antibodies was then performed: live/dead blue dye (Invitrogen), anti-CD8 Amcyan (BD Biosciences), anti-CD3 APC-Cy7 (Biolegend), anti-PD-1 PercpCy5.5 (BD Biosciences), anti-CTLA4 PE-Cy7, anti-CD244 FITC, and anti-CD160 PE (Biolegend) before washing and flow cytometric analysis. Memory subset analysis utilized the same panel but included anti-CCR7 FITC (R&D systems) and anti-CD45RA AF700 (Biolegend) and omitted anti-CTLA4, anti-CD244, and anti-CD160. Example flow plots can be found in (Figure S1).

### Peptide Stimulation Assays

Following identification of CMV positive and negative donors, 2 × 10^6^ cells were incubated at 37°C for 5 h with either PMA and ionomycin, CMV peptide mix (10) or EBV peptide mix at 10 μg/ml (Supplementary Table 6), along with protein cocktail inhibitor mix (eBiosciences). Live/dead red dye (Invitrogen), anti-CD3 APC-Cy7 (Biolegend) anti-CD8 Amcyan (BD Biosciences), were then applied before fixation and permeabilisation and IFN-γ AF700 (Biolegend) and TNF-α PE-Cy7 staining (eBioscience). Example flow plots can be found in supplementary (Figure S1). For assessing immune cell activation and cytotoxic degranulation, 2 × 10^6^ cells were stimulated with either the above peptide mixes overnight at 37°C or a cell stimulation cocktail (Invitrogen) for 5 h. At the time of stimulation, CD107a FITC (Biolegend), along with brefeldin A and monensin was incorporated into the stimulation panel (example staining of CD107a can be found in supplementary).

### Statistical Analysis

Mann-Whitney or Kruskal-Wallis testing for comparisons and multiple regression models were performed.

## Results

**Figure 1:**
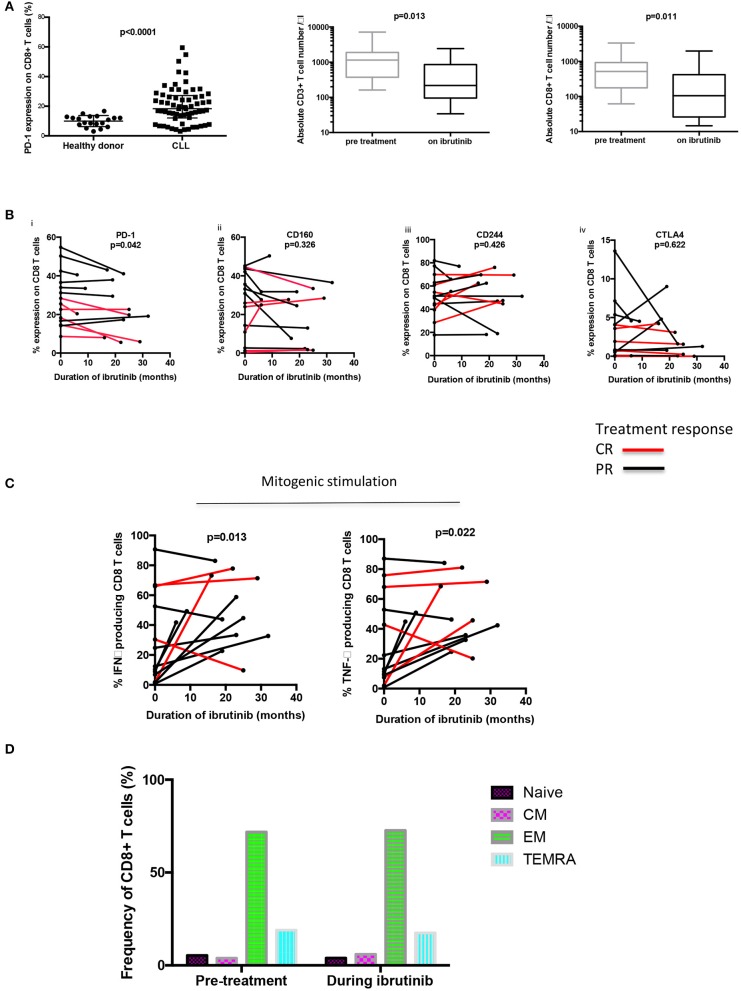
Long term ibrutinib therapy decreases PD-1 expression on CD8+ T cells and increases the functional response to mitogen stimulation. **(A)** PD-1 expression on CD8+ T cells was ascertained using flow cytometry. An increased frequency of PD-1 expression was observed amongst untreated patients and those treated with chemo-immunotherapy only (*n* = 65), compared to healthy donors (*n* = 19). A reduction in the absolute number of both CD3+ and CD8+ T cells was observed during long term ibrutinib therapy. **(B)** The frequency of expression of checkpoint receptors on CD8+ T cells of 14 patients with relapsed refractory CLL treated with ibrutinib is shown over the treatment duration, including (i) PD-1, (ii) CD160, (iii) CD244, and (iv) CTLA4). A decreased percentage of PD-1 positive CD8+ T cells was observed in the patients with CLL during long-term ibrutinib therapy. **(C)** PBMCs from 13 patients with CLL were stimulated with PMA plus ionomycin, before and during ibrutinib therapy. The CD8+ T cells producing IFNγ and TNFα were identified through intracellular staining and flow cytometric analysis and an increased frequency of both cytokine-producing CD8+ T cells were found in B-CLL patients during ibrutinib therapy. **(D)** Memory subset analysis was performed using CCR7 and CD45RA to define naïve, central memory (CM), effector memory (EM), and T_EMRA_ CD8+ T cell populations. No difference in the frequency of the subsets of memory cells was found before or during ibrutinib therapy (*n* = 4).

Amongst untreated patients (*n* = 42), and those previously treated with chemo-immunotherapy only (*n* = 23), 18.3% of CD8+ T cells expressed PD-1, an increased frequency compared to healthy age-matched donors (10.8%; *p* = 0.0001) (Figure 1A). No association was observed in relation to previous treatment with chemo-immunotherapy (Figure S2).

Amongst patients treated with ibrutinib [median 21 months (range 6–32)] the CD3+ T cell count was substantially reduced [median 1,154 cells/μl to 216 cells/μl; (*p* = 0.013)] and the CD8+ T cell count also decreased markedly from median 515 cells/μl to 104 cells/μl; (*p* = 0.011). As expected, the total lymphocyte count fell from 25 to 3.4 × 10^9^/l during the treatment period (Figure 1A).

Interestingly the use of ibrutinib was associated with a reduction in PD-1 expression on CD8+ T cells [28% pre-treatment vs. 24.6% (*p* = 0.042)]. In addition, patients who reached a complete response (CR) as defined by IWCLL criteria, had a greater delta change in their PD-1 expression compared to those obtaining a partial response (−0.25 vs. −0.03; *p* = 0.043) (11). Patients who achieved a CR with ibrutinib treatment, also tended to have a lower frequency of PD-1 CD8+ T cells prior to commencing therapy, although this did not reach statistical significance (24.05 vs. 35.3%; *p* = 0.130). Importantly the duration of ibrutinib therapy was not found to differ between patients who achieved PR compared to those reaching a CR (23.5 vs. 21 months, respectively; *p* = 0.924) and no difference was seen in expression of other inhibitory markers that are increased in patients with CLL (1) (CD244 (52 vs. 55%; *p* = 0.426), CD160 (25 vs. 23%; *p* = 0.326), or CTLA4 expression (3.05 vs. 2.55%; *p* = 0.622) (Figure 1B).

We next went on to examine the functional activity of T cells and initially stimulated PMBC with PMA and ionomycin mitogen. Serial samples from patients on ibrutinib exhibited a 3.5 fold increase during therapy in the proportion of CD8+T cells that produced TNFα and INFγ following mitogenic stimulation [TNFα 13.1–45.7% (*p* = 0.013); IFNγ 12.4–44.7% (*p* = 0.0215) *n* = 13] (Figure 1C). However, the absolute number of cytokine-positive CD8+ T cells remained unchanged [pre-treatment IFNγ producing CD8+ T cells: 144 vs. 311 cells/μL during ibrutinib (*p* = 0.07) and TNFα producing CD8+ T cells: 147 pre-treatment vs. 306 cells/μL during ibrutinib (*p* = 0.09)] suggesting that long-term ibrutinib therapy acts to reduce the frequency of hypofunctional T cells. To address the impact of ibrutinib on T cell cytotoxicity, PBMCS were incubated with a T cell stimulation cocktail and CD107a degranulation assessed. Although no statistical difference in the frequency of response was noted, a trend towards increased CD107a release was observed with therapy [pre-ibrutinib 11.1% vs. 24.2% during ibrutinib (*p* = 0.485; n = 4)]. To assess if ibrutinib therapy impacted on the memory status of CD8+ T cells, a comparison was made before and during therapy. However, no difference in the frequency of memory cell subsets of CD8+ T cells was found (2 way Anova of repeating measures *p* = 0.998; *n* = 4) (Figure 1D).

The impact of ibrutinib on antigen-specific immune responses was next investigated through the use of HLA-peptide tetramer staining and viral peptide stimulation. Donor CMV and EBV serostatus and HLA genotype was first determined. Following incubation overnight with pooled CMV or EBV peptide, CD8+ T cell release of CD107a was assessed and compared between samples taken before and during ibrutinib therapy. No difference was observed in the release of CD107a following antigen stimulation [21.34% before therapy vs. 22.16% during therapy (*p* = 0.879)].

**Figure 2:**
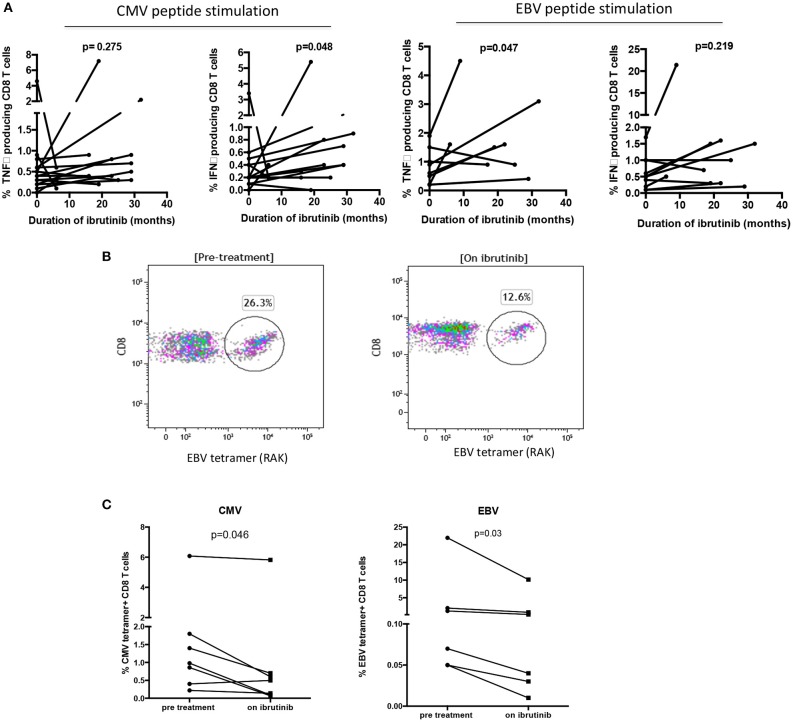
Long term ibrutinib therapy decreases the frequency of virus-specific CD8+ T cells and improves the functional response to stimulation with viral peptides. PBMCs from patients with CLL were stimulated with pooled CMV and EBV peptides. The CD8+ T cells producing IFNγ and TNFα were identified through intracellular staining and flow cytometric analysis. The percentage of cytokine producing CD8+ T cells were compared between patients before and during ibrutinib therapy. **(A)** (i) With pooled CMV peptide stimulation, significantly increased frequencies of IFNγ producing CD8+ T cells were found in patients with CLL during ibrutinib therapy (*p* = 0.048). (ii) With pooled EBV peptide stimulation, significantly increased frequencies of TNFα producing CD8+ T cells were found in patients during ibrutinib therapy (*p* = 0.047). **(B)** An example of the flow cytometric plot of EBV tetramer staining is shown, demonstrating the reduced frequency of EBV specific CD8+ T cells during ibrutinib therapy. **(C)** The frequency of CMV specific CD8+ T cells and EBV-specific CD8+ T cells in B-CLL patients before and during ibrutinib therapy were compared. The frequencies of both virus-specific cells decreased during ibrutinib treatment in B-CLL patients (*p* = 0.046 for CMV and *p* = 0.03 for EBV).

Next, the appropriate HLA class I tetramer staining of PBMC was combined with surface membrane immunophenotyping. PD-1 expression on CMV-specific CD8+ T cells was not found to be modulated by ibrutinib therapy (12.6 vs. 11.1%; data not shown) suggesting that CMV-specific CD8+ T cells do not account for the reduced frequency of PD-1 positive cells observed in the total CD8+ T cell population. However, paired PBMC samples showed an increased frequency of cytokine production with BTKi treatment following CMV-peptide pool stimulation [IFNγ: 0.46–0.78% (*p* = 0.048) and TNFα: 0.69–1.05% (*p* = 0.274)] (Figure 2A). The frequency of cytokines produced by EBV-specific CD8+ T cells also increased [TNFα: 0.85–1.81% (*p* = 0.047) and IFNγ 0.63 vs. 2.34% (*p* = 0.219)] (Figure 2A). Despite this, no difference was found in their absolute number before and during ibrutinib therapy [CMV peptide stimulation: (IFNγ: 5.6 vs. 5.8 cells/μL (*p* = 0.56); TNFα 2.2 vs. 3.7 cells/μL (*p* = 0.3) and EBV peptide stimulation: IFNγ: 0.85 vs. 1.8 cells/μL (*p* = 0.05); TNFα 0.63 vs. 3.2 cells/μL (*p* = 0.22)]. This indicates that the frequency but not absolute number of hypofunctional EBV or CMV-specific CD8+ T cells is reduced during ibrutinib therapy.

We were further interested to see if this improved functional activity might lead to a reduction in the number of virus-specific T cells. Indeed, HLA-peptide tetramer staining showed that the median frequency of CMV-specific T cells fell from 1.7% of the CD8+ repertoire before ibrutinib to 1.1% during therapy (*p* < 0.05; *n* = 7). Similarly, EBV-specific responses were also reduced by over 50% (4.2% pre-ibrutinib vs. 1.9% during therapy; *n* = 6) (Figures 2B,C).

## Discussion

Ibrutinib has transformed the management of CLL and many patients have now been on continuous therapy for many years. However, despite proven efficacy in suppression of B cell lymphoproliferation little is known regarding the impact of ibrutinib on immune function. Our analysis determined the impact of ibrutinib on antigen-specific T cells for the first time and also assessed patients with the longest treatment duration to date (9).

The striking reduction in CD3+ and CD8+ T cell number during ibrutinib therapy has been observed previously (12). In contrast Long et al. noted that the total CD8+ T cell response actually increased during ibrutinib therapy but this was within the first 6 months of therapy, when the absolute lymphocyte count was almost double the initial pre-treatment lymphocyte count. The difference observed between this work and the work of Long et al., may reflect a difference in the duration of ibrutinib therapy as similarly to our findings, Niemann et al. reported a reduction in T cell numbers by week 48 of ibrutinib treatment (13). Increased expression of PD-1 on CD8+ T cells is a characteristic feature of patients with CLL and predicts progression risk (1, 3). Importantly, long-term ibrutinib therapy reduced PD-1 expression on CD8+ T cells and this effect was not observed following conventional chemotherapy. An intriguing observation was that the reduction in PD-1 expression was more pronounced in patients who went on to achieve a complete remission in response to ibrutinib therapy and this group also exhibited a trend towards a lower overall percentage of PD-1+ CD8+ T cells prior to therapy. It is currently unclear if this correlation reflects a secondary improvement in immune function within individuals who gain excellent clinical responses to ibrutinib or if reversal of T cell exhaustion may itself play a role in mediating the therapeutic response to ibrutinib treatment.

The expression of additional checkpoint proteins was not modulated by ibrutinib therapy. Expression of intracellular CTLA4 has previously been reported to decrease with ibrutinib therapy, whereas our analysis assessed surface expression staining (10). PD-1 is a defining phenotypic feature of T cell exhaustion and we observed increased cytokine responses within CD8+ cells following long-term ibrutinib therapy indicating that BTK inhibition also reverses features of functional exhaustion. This may be achieved partly through suppression of the CLL clone, which shares features with B regulatory cells and correction of a range of elevated cytokines is observed within the first 2 months of ibrutinib therapy (12, 14). Further mechanisms for reversal of T cell exhaustion may include a reduction in chronic antigenic stimulation both from the decrease in tumour load and improved immune competence against infective agents.

To evaluate the latter we focused on the immune response to latent herpesviruses, which drive expanded CD8+ T cell responses in CLL in a mechanism that is thought to reflect a response to increased endogenous viral replication. EBV infection is associated with accelerated time to disease progression in CLL (15) although CMV has no known deleterious effect (16). Of interest, the magnitude of CD8+T cell CMV-specific responses increased with advanced stage disease, in line with a previous study of CD4+ immunity (data not shown) (17). Importantly, the magnitude of the virus-specific immune responses reduced during ibrutinib therapy, with a comparable increase in peptide-specific cytokine responses. These findings are the first report of improvement in antigen-specific immune responses following ibrutinib therapy. The proportion of PD-1+ CMV-specific T cells was not influenced by ibrutinib therapy, despite a decrease in the global PD-1+ CD8+ pool, indicating that reversal of T cell exhaustion may be directed towards tumour specific T cell responses. This also suggests that PD-1 is not contributing to the improvement in antigen-immune response observed in virus-specific T cells. Indeed, previous published work found a normal cytokine response in CMV-specific CD8+ T cells in patients with CLL, when CMV peptide was presented via lymphoblastoid cell lines or healthy donor B cells and in a controlled B: T cell ratio. In contrast, cytokine responses were impaired when CMV peptide was presented via CLL cells. The improvement observed in herpes-virus specific T cells in this study may therefore relate to the reduction in the CLL clone, rather than the expression of PD-1 (18).

Inducible T cell kinase (ITK) plays an important role in the maintenance of Th2 CD4+ cells and memory CD8+ T cells and is known to be inhibited by ibrutinib (5). However, Itk^−/−^ CD8+ memory T cells (in comparison to naïve CD8+ T cells) demonstrate normal recall responses to bacterial infection in terms of frequency and functionality and this is compatible with our findings (19). The impact of ibrutinib on the induction of primary immune responses mediated by naïve T cells deserves further investigation. Ibrutinib has previously been associated with improvements in T cell function including an increase the degree of diversity within the T cell repertoire (14) and enhanced outcome of CAR-T therapy in patients with CLL (20). Our findings now demonstrate a reversal in the degree of phenotypic and functional exhaustion and help to explain the encouraging clinical experience of BTKi therapy in relation to infection risk (21).

## Data Availability Statement

The raw data supporting the conclusions of this article will be made available by the authors, without undue reservation, to any qualified researcher.

## Ethics Statement

The studies involving human participants were reviewed and approved by Ethical approval was obtained from West Midlands regional ethics committee for patients (10/H1206/58) and for healthy donor controls (2002/073). The patients/participants provided their written informed consent to participate in this study.

## Author Contributions

HP and HL designed the research. HP, NM, JB, NC, JZ, MK, and CO conducted the experiments. CH performed statistical analysis. SP, MK, and TM recruited patients and followed patients up. HP, GP, TS, and PM wrote the manuscript.

### Conflict of Interest

The authors declare that the research was conducted in the absence of any commercial or financial relationships that could be construed as a potential conflict of interest.
