# Supplementary material for: Long-Term Ibrutinib Therapy Reverses CD8+ T Cell Exhaustion in B Cell Chronic Lymphocytic Leukaemia
Source: Front Immunol. 2019 Dec 12;10:2832. doi: 10.3389/fimmu.2019.02832 (PMC6921985; doi:10.3389/fimmu.2019.02832)
Supplement: Supplementary file 1 [file Data_Sheet_1.docx]

Supplementary

**Table 1:**

**Table 2:**

**Table 3:**

Table 4:

***Complete response****: Lymphocyte count <4 x 10^9^/L, absence of lymphadenopathy and hepatosplenomegaly on examination, no constitutional symptoms and platelet >100x10^9^/L , neutrophils >1.5x10^9^/L and Hb> 110g/L.* ***Partial response:*** *≥50% improvement in lymphadenopathy, or hepatosplenomegaly or lymphocyte count from baseline*

**Table 5:** EBV and CMV HLA-class 1 tetramers

**EBV tetramers CMV tetramers**

**Table 6:** EBV peptide pool mix1:

| **HLA** | **Epitope** | **Protein** |
| --- | --- | --- |
| A2 | FLYALALLL | LMP2 |
|  | CLGGLLTMV | LMP2 |
|  | YVLDHLIVV | BRLF1 |
|  | GLCTLVAML | BMLF1 |
|  | LLIEGIFFI | BaRF1 |
| B7 | RPQKRPSCI | EBNA1 |
|  | IPQCRLTPL | EBNA1 |
|  | RPPIFIRRL | EBNA3A |
|  | QPRAPIRPI | EBNA3C |
|  | RPRATWIQEL | BaRF1 |
|  | RPGRPLAGFYA | BNLF2b |
|  | RPQGGSRPEFVKL | BMRF1 |
| B8 | QAKWRLQTL | EBNA3A |
|  | FLRGRAYGL | EBNA3A |
|  | RAKFKQLL | BZLF1 |
|  |  |  |

**Figure S1: example of staining flow plots of CD8+ and tetramer identification, memory status, CD107a, IFN-γ, TNF-α, CD160, CTLA4, CD244 and PD1,**

**Figure S2:** The expression of PD-1 on CD8+T cells according to clinical and prognostic variables in patients with CLL and following ibrutinib therapy

1a) The percentage of PD-1 positive CD8+ T cells within the 65 B-CLL patients (untreated and those treated with conventional chemo-immunotherapy) were compared according to i) stage of disease, ii) IGVH mutation status, iii) CD38, iv) FISH results, v) treatment history and vi) age using multivariate analysis. No significant difference was found between these variables, except the age group, which showed a higher percentage of PD-1 positive CD8+ T cells in the patients aged 70-80 (23.1%), compared to those less than 70 (8%) and 80+ (14.2%; p<0.05).

**
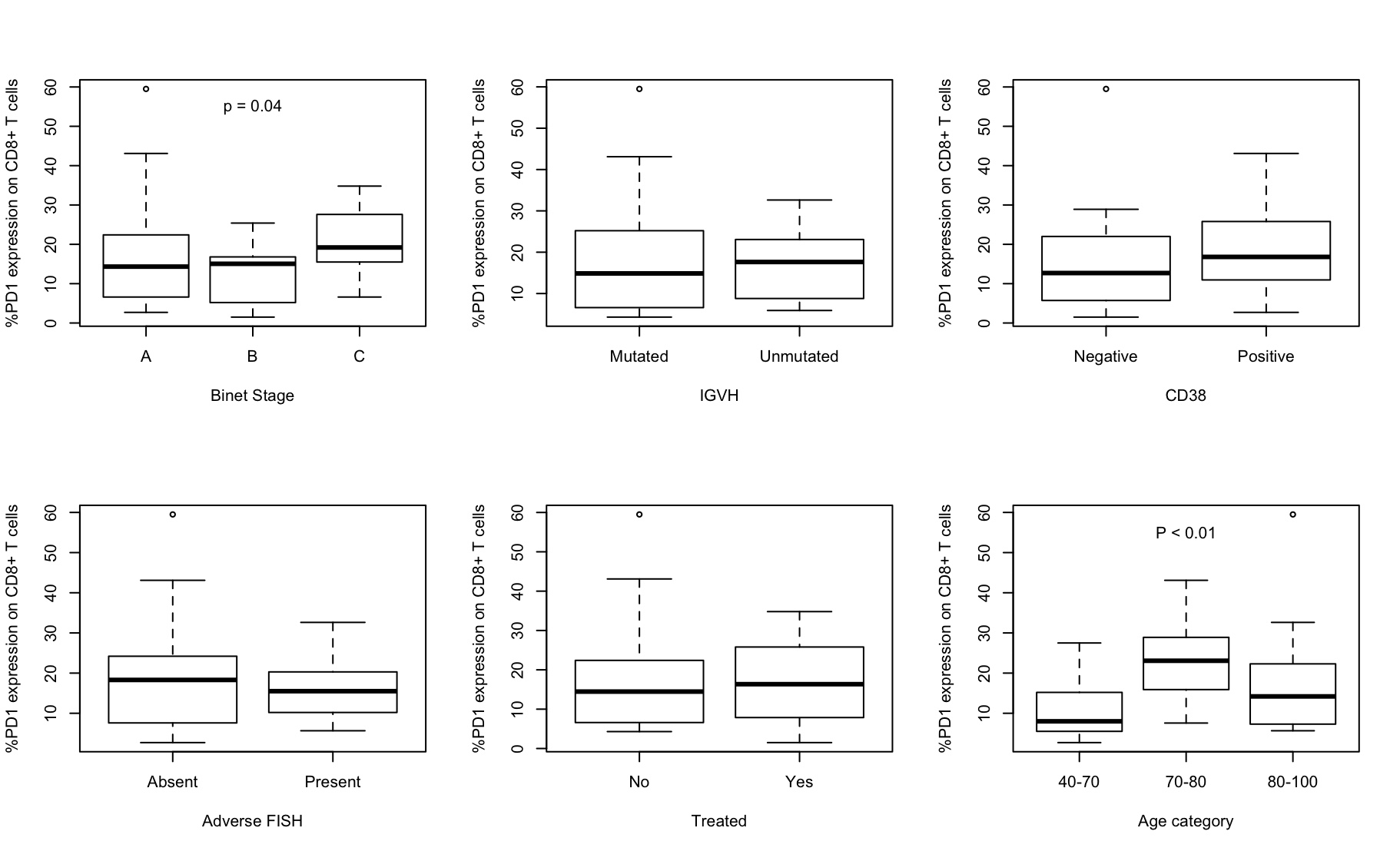
**
